# Account of a Remarkable Hæmorrhagic Disposition, Existing in Many Individuals of the Same Family

**Published:** 1815-01

**Authors:** John Hay

**Affiliations:** of Reading, Fellow of the Massachusetts Medical Society.


*For the Medical and Physical Journal.*

*Account of a remarkable Hæmorrhagic Disposition, existing in many Individuals of the same Family;* by Dr. JOHN HAY, of Reading, Fellow of the Massachusetts Medical Society.

THE first person of whom I have any account, as being subject to the remarkable predisposition to be described, was Mr. Oliver Appleton of Ipswich, about one hundred years since. This man was subject from his youth to profuse bleeding from slight causes. When advanced in life, by a long confinement in bed the skin was worn from his hips, and an hæmorrhage taking place from that part and from the urethra, occasioned his death. He left three daughters, two of whom married into a family by the name of Swain, in Reading. Dr. Thomas Swain, who married the eldest, had by her two sons and five daughters. The two sons both bled to death. During their lives they were liable to violent hæmorrhages from the smallest scratch or injury, the bleeding usually coming on about a week after they were hurt.

Dr. Oliver Swain, July 17, 1770, met with an injury from the kick of a horse, which laid the leg open to the bone, three inches in length. He bled profusely for some hours, suffering extreme pain. After this, there was no farther effusion of blood till Wednesday 21st, at twelve, at which time the part began, after dressing, to bleed a little. On Monday, 26th, it was dressed again, but bled a little. Tuesday evening, 27th, bled somewhat. But on Wednesday evening, at eight o'clock, it began to bleed fast, and continued to do so two or three hours. On Thursday, 29th, after dressing, bled but little; at eight the hæmorrhage came on again, attended with great pain, but went off soon. Friday, 30th, small hæmorrhage, all the fore part of the day, with extreme pain. At twelve, began to bleed very fast, and continued bleeding three or four hours; after which time he grew delirious, and remained so till evening, then was more rational, but threatened with fever, notwithstanding his great loss of blood. 31st, in the morning, more comfortable for a short time; he soon, however, lost his senses, and about noon his speech, and in the evening was thought dying. August 1st, he lay in a restless situation, senseless and speechless, a small bleeding attending. At evening, the bleeding was more profuse, but stopped after dressing. Monday, 2d, lay all day, without speaking, until night; he then spoke a few words. Tuesday, 3d, more comfortable, rational, and had his speech, though somewhat bewildered. Dr. Amos Putman and Dr. Thomas Swain, jun. dressed his wound, and found the tibia bare for the size of a cent, but in a good way. Wednesday, 4th, much worse in the morning; in the afternoon, delirious and stupid. Thursday, 5th, very low the fore part of the day, had poultices put to his feet; in the afternoon, he had a return of his senses, and was more comfortable than he had been for some days. Friday, 6th, very ill in the morning, greatly distressed for breath. Between eight and nine o'clock, grew much worse, and continued in the greatest distress of body, though somewhat composed in mind, until about one o'clock, when he died, aged 33 years.

Dr. Thomas Swain, jun. having received a small wound on his finger, with a pen-knife, the hæmorrhage reduced him almost to death. Soon after he became able to ride out, he was attacked with bleeding at his lungs, that closed the scene at the age of thirty.

Gen. Benjamin Brown married the oldest daughter of Thomas Swain, jun. had by her three sons, two of them bleeders, (for this is the distinction they go by,) one of them bled to death at the age of fifteen years; the other, now about the age of fifty-four, is living in this neighbourhood. He informed me, a few days since, that he bruised his right hand the last winter, 1810; and, although he broke no skin, the blood some time after burst from the bruise, and continued to flow for a fortnight. He supposed he lost two quarts of blood. He has three three daughters, and thinks they are in the proper line for bleeders. Age does not exempt them from this disposition. Two of the daughters married into the family of the Nortons; one of them had three bleeders, the other had two; one of these bled to death, the other was drowned. Mr. John Bachilor married a daughter of Dr. Thomas Swain, sen. who had three bleeders, two of whom are dead; the one that is living bled profusely the last winter, 1810. The children of bleeders are never subject to this disposition, but their grandsons by their daughters are.

Dr. Oliver Swain has three sons that are living with us, none of whom are subject to bleeding. Dr. Oliver Swain left one daughter, married to Jeremiah Hartshorn, my next-door neighbour, who has three bleeders; the oldest of them, from a small scratch by a plaything, when nine months old, bled nine days, notwithstanding the application of styptics, astringents, and bark, with preparations of nitre, which were of no avail. The second son has also bled from time to time.

May 5th, 1810, the third son, who is about eight years of age, received a small wound upon two fingers of the left hand, hæmorrhage occurred from one of them only. It has been observed always to be the case, that, though many wounds are made, only one bleeds, and the bleeding continues till nothing but a serous fluid issues from it. This patient bled for four days, and the more he bled, the pulse was found to grow more rapid. The countenance was pale and ghastly.

Dr. John Otto has given an account, which I have consulted, of an hæmorrhagic disposition existing in two families, of the names of Smith and Shepherd, living in the vicinity of Plymouth, New-Hampshire. A person by the name of Appleton, married a Smith of Haverhill, supposed to be the Mrs. Smith mentioned by Dr. Otto.

I have attended three or four persons of this hæmorrhagic disposition, and have found no article answer better than the sulphate of soda, used as follows:

℞. Sulphat. Sodæ. unciam unam.

This is to be administered for two or three days in succession, and generally stops the hæmorrhage. A more frequent repetition of the medicine is certain of producing the effect. This practice was very beneficial in the case of Mr. Hartshorn's child, a constipation of the bowels being a never failing evil among them, and vomiting very harassing.

Thomas Swain, junior, left one daughter and one son; the daughter married Mr. Parker, of Reading. She had two bleeders. One of them died with an hæmorrhage about a year since. The other bled profusely the last winter, 1810. Benjamin Swain married the second daughter of Oliver Appleton.

She had one bleeder only, although they had two sons and one daughter. The bleeder died with the throat distemper. Mrs. Whipple had but one son, of a dark complexion, which is not usual at all with those who are subject to this indisposition, for they are of a florid countenance, are remarkably healthy, and extremely irascible.

Dr. Rush has been consulted twice in cases similar to these, in the course of his practice. The first time by a family in York, and the other by one in Northampton county. A. B. of the state of Maryland, has had six sons; four of them died with the loss of blood from the most trifling scratches or bruises. A small pebble fell on the fore finger nail of one of these children when at play, being one or two years old; in a short time the blood issued from the end of the finger until he died. The physicians could not stop the bleeding. Two of the brothers are now suffering from the same hæmorrhagic disposition; they bleed profusely from the slightest scratch; and the father looks every day for an accident that will destroy them. Ligatures have been applied above the wounds, and a bladder secured over the parts, by the direction of Dr. Brattle of Cambridge, in a former bleeding of Dr. Oliver Swain; the blood has burst out above the ligatures, which has caused extreme distress. It has been observed of these bleeders, that, although they may not fall a sacrifice to the hæmorrhage, their lives are of short continuance. Nathaniel Brown, now living in this town, is said to be the oldest person of any of the bleeders since Mr. Oliver Appleton. My oldest son, Jonathan P. Hay, married a descendant of Mr. Appleton, and has eight children by her; three sons, the youngest about eight years old, and has the exact complexion of the bleeders, but as yet has not bled more than common. I am apprehensive that he will exhibit the hæmorrhagic disposition. The great grandmother of Mr. Hartshorn's children pronounced them bleeders in their infancy; her predictions, with regard to her bleeding descendants, have always been verified.—*New-England Journal.*

